# 1494. Impact of Polypharmacy on Gait Speed and Falls in Older People Living with HIV.

**DOI:** 10.1093/ofid/ofad500.1329

**Published:** 2023-11-27

**Authors:** Priya Kosana, Kunling Wu, Katherine Tassiopoulos, Scott Letendre, Qing Ma, Kristine M Erlandson, Shelli F Farhadian

**Affiliations:** Yale School of Public Health, Waxhaw, North Carolina; Harvard University T H Chan School of Public Health, Boston, Massachusetts; Harvard University T H Chan School of Public Health, Boston, Massachusetts; University of California, San Diego, California; University at Buffalo, Buffalo, New York; University of Colorado Anschutz Medical Campus, Aurora, CO; Yale School of Medicine, New Haven, Connecticut

## Abstract

**Background:**

Older people living with HIV (PWH) are uniquely prone to polypharmacy because of the increased medication burden associated with antiretroviral drug use and higher rates of medical comorbidities in this population, leading to more medications prescribed. We assessed for the association between polypharmacy and hyperpolypharmacy with two adverse geriatric outcomes: slow gait speed and falls.

**Methods:**

In this cross-sectional analysis we leveraged entry visit clinical data from the AIDS Clinical Trials Group (ACTG) A5322 (HAILO) cohort of older PWH. We included HAILO study participants with plasma viral load < 200 copies/ul and either gait speed or falls assessments. Polypharmacy was defined by receipt of ≥ 5 prescription medications (including antiretroviral therapy); hyperpolypharmacy was defined as ≥ 10 medications. We fit logistic regression models for slow gait speed (< 1 meter/second) and multinomial logistic regression models for single and recurrent (≥ 2) falls in the past 6 months, and adjusted for sex, age, self-reported race, and number of comorbidities. We explored differences in associations by sex by including interaction terms in the models.

**Results:**

Nine hundred seventy-seven participants were included, with demographic and clinical information in Table 1. Forty-four percent of participants had polypharmacy, 8% had hyperpolypharmacy. Women were more likely to experience polypharmacy than men (61% vs 46%; p < 0.01). Black women were disproportionately prescribed opioids (21% vs. 10%, p< .001). Polypharmacy and hyperpolypharmacy were associated with higher odds of slow gait speed (Figure 1) and with single and recurrent falls (Figure 2). There were no differences by sex in the associations between polypharmacy and hyperpolypharmacy and either outcome.

Demographic and Clinical Information of Study Participants

**Figure:**
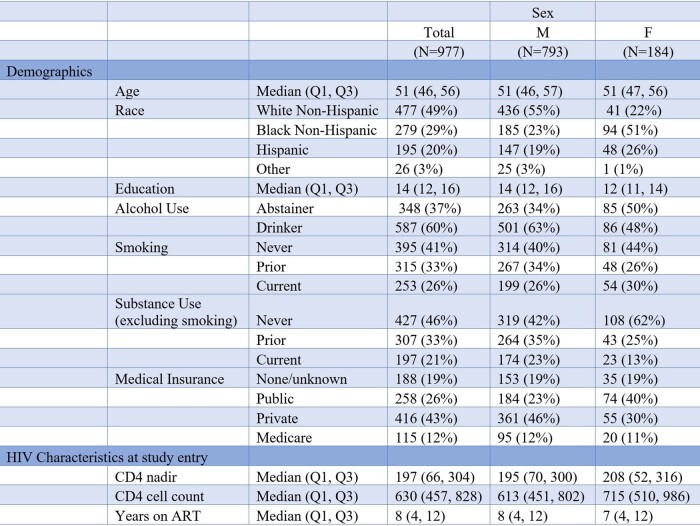

The demographic and clinical characteristics of the study participants is listed in Table 1. Race, years of education, alcohol use, other substance use, and medical insurance were all significantly different (p < 0.05) among the male and female groups. Men in the cohort have a higher CD4 count, have been on ART treatment longer, and have a lower CD4:CD8 ratio than women at study entry.

Increased Odds of Slow Gait speed with Polypharmacy and Hyperpolypharmacy

**Figure 1:**
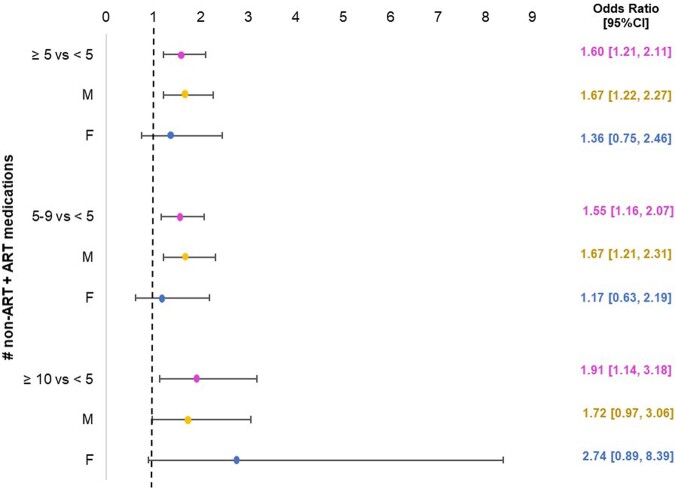
includes all prescription medications, including ART. Polypharmacy and hyperpolypharmacy were associated with slow gait speed (< 1 meter/second) when compared to non-polypharmacy (polypharmacy odds ratio (OR) = 1.60 [1.21, 2.11], hyperpolypharmacy OR = 1.91 [1.14, 3.18]).

Increased Odds of Recurrent falls with Polypharmacy and Hyperpolypharmacy

**Figure 2:**
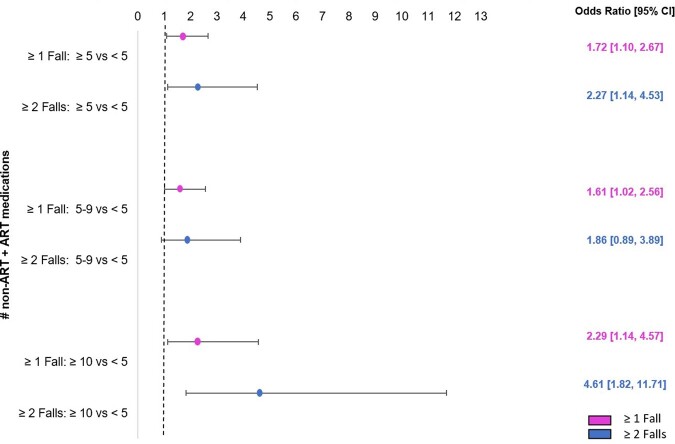
includes all prescription medications, including ART. Experiencing 2 or more falls in the prior 6 months was associated with polypharmacy (OR = 2.27 [1.14, 4.53]) and hyperpolypharmacy (OR = 4.61 [1.82, 11.71]).

**Conclusion:**

In older PWH, polypharmacy and hyperpolypharmacy were associated with slow gait speed and recurrent falls, even after accounting for medical comorbidities. These results highlight the need for increased focus on the risks and management of polypharmacy in PWH.

**Disclosures:**

**Kristine M. Erlandson, MD MS**, Gilead: Advisor/Consultant|Gilead: Grant/Research Support|Merck: Advisor/Consultant|VilV: Advisor/Consultant

